# P-1933. Co-Administration of Triazoles with Chemotherapy and/or Immunosuppressants Known to Have Moderate-to-Severe Drug-Drug Interactions in Patients with Hematologic Malignancies Who Are Hospitalized for Invasive Aspergillosis

**DOI:** 10.1093/ofid/ofaf695.2101

**Published:** 2026-01-11

**Authors:** Thomas J Walsh, Craig I Coleman, Melissa D Johnson, Belinda Lovelace, Barbara D Alexander

**Affiliations:** Center for Innovative Therapeutics and Diagnostics (citdx.org), Richmond, VA; University of Connecticut School of Pharmacy, Storrs, CT; Duke University, Durham, North Carolina; F2G, Inc., Princeton, New Jersey; Duke University School of Medicine, Durham, North Carolina

## Abstract

**Background:**

Chemotherapy and immunosuppressant use in patients with hematologic malignancies increases their risk of invasive aspergillosis (IA). Antifungal triazoles are used for treatment of IA but can cause serious drug-drug interactions (DDIs) with chemotherapies and immunosuppressants via inhibition of CYP3A4. The extent of these DDIs in treatment of IA is unknown in real world settings.

**Figure:**
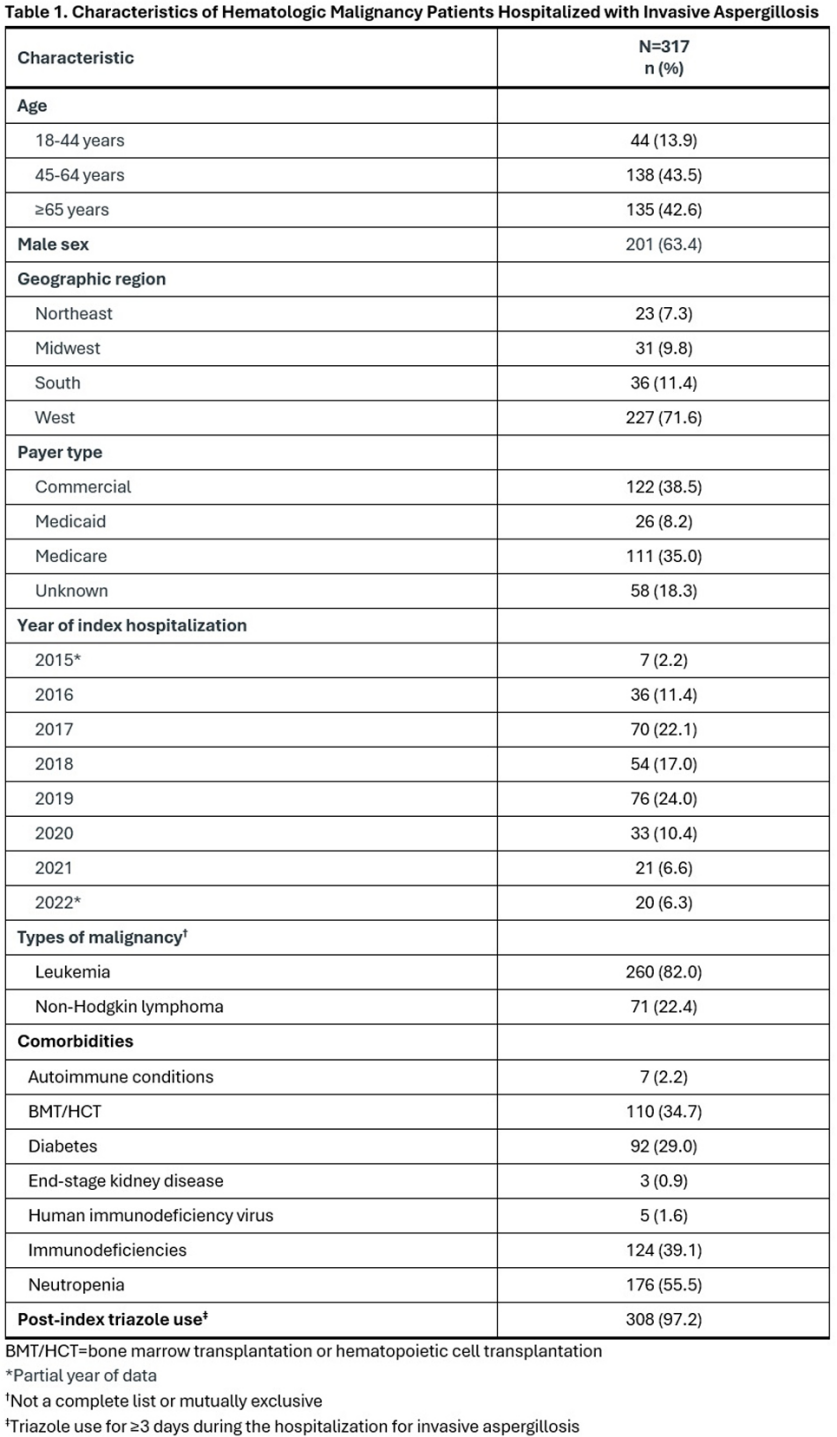

**Figure:**
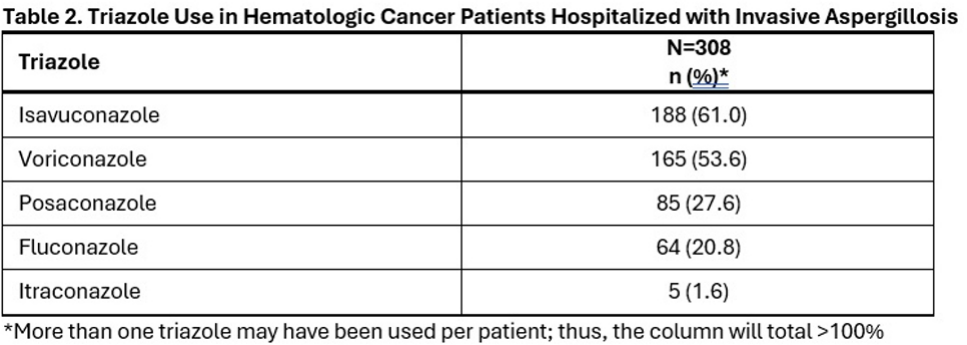

**Methods:**

We studied US IQVIA claims including adults with ≥1 claim for an inpatient stay with a new diagnosis code for IA (B44.0, B44.1, B44.2, B44.7) from October 2015-November 2022 and evidence of systemic antifungal therapy for ≥3 days during the hospitalization. The cohort was limited to patients with recent hematologic malignancy defined by the presence of ≥1 claim with a diagnosis code of C81-C96 within 6 months prior to IA admission (index date). The proportion of patients receiving a triazole with chemotherapy and/or an immunosuppressants known to have moderate-to-severe DDI was determined.

**Figure:**
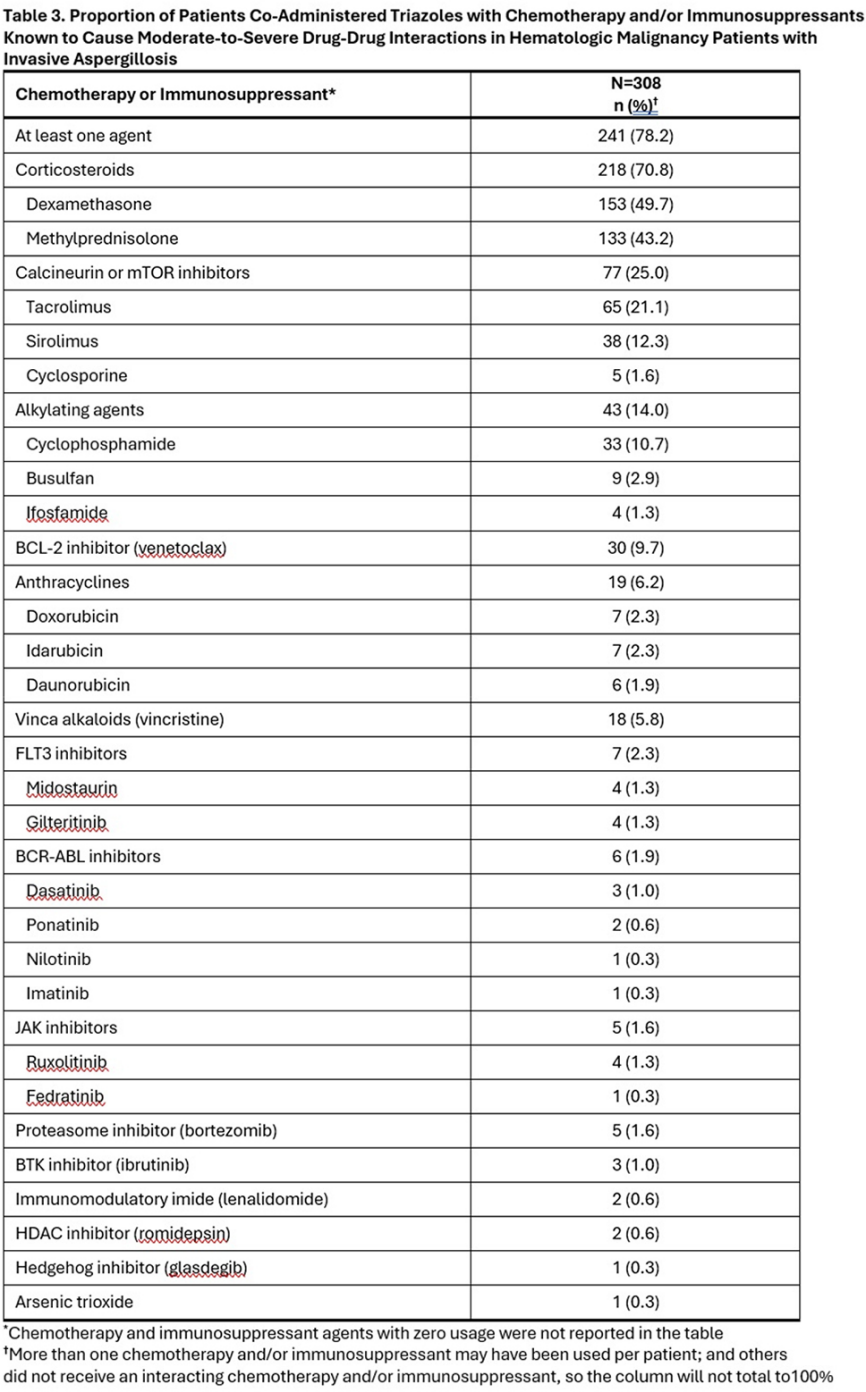

**Results:**

Triazoles, mostly isavuconazole (61.0%) and voriconazole (53.6%), were used in 308 of 317 (97.2%) patients for a mean ± standard deviation of 146 ± 217 (median = 64) days after IA admission (Tables 1 and 2). Of these, 241 (78.2%) received an interacting chemotherapy and/or immunosuppressant known to have a moderate-to-severe DDI with triazoles (Table 3). The most frequent chemotherapy or immunosuppressants administered with a triazole included corticosteroids (70.8%), calcineurin or mTOR inhibitors (25.0%), alkylating agents (14%), BCL-2 inhibitors (9.7%), anthracyclines (6.2%) and vinca alkaloids (5.8%).

**Conclusion:**

Co-administration of triazoles potentially interacting with chemotherapy or immunosuppressants occurred in most (97.2%) patients with hematologic malignancies and IA. To reduce serious adverse events, therapeutic drug monitoring and/or dose adjustment of chemotherapy or immunosuppressants may be warranted. Antifungal agents without potentially serious DDIs with chemotherapy or immunosuppressants are needed for treatment of IA in patients with hematologic malignancies.

**Disclosures:**

Thomas J. Walsh, MD, PhD (Hon), FIDSA, FAAM, FECMM, Allergan: Grant/Research Support|Astellas: Advisor/Consultant|Astellas: Grant/Research Support|Basilea: Advisor/Consultant|F2G Inc.: Advisor/Consultant|F2G Inc.: Grant/Research Support|Gilead: Advisor/Consultant|Gilead: Grant/Research Support|Karyopharm: Advisor/Consultant|Lediant: Advisor/Consultant|Lediant: Grant/Research Support|Merck: Advisor/Consultant|Merck: Grant/Research Support|Partner Therapeutics: Advisor/Consultant|Scynexis: Advisor/Consultant|Scynexis: Grant/Research Support|Shionogi: Advisor/Consultant|Shionogi: Grant/Research Support|Statera: Advisor/Consultant|T2 Biosystems: Advisor/Consultant|T2 Biosystems: Grant/Research Support|Viosera: Grant/Research Support Craig I. Coleman, PharmD, F2G Inc.: Advisor/Consultant|F2G Inc.: Grant/Research Support Melissa D. Johnson, PharmD MHS AAHIVP, Biomeme: Licensed technology, method to detect fungal infection|Biomeme: Licensed technology, method to detect fungal infection|Scynexis: Grant/Research Support|Scynexis: Grant/Research Support|UpToDate: Author Royalties|UpToDate: Author Royalties Belinda Lovelace, PharmD, MS, MJ, F2G Inc.: Employee

